# Hybrid Glenoid Designs in Anatomic Total Shoulder Arthroplasty: A Systematic Review

**DOI:** 10.1177/15563316211040829

**Published:** 2021-09-03

**Authors:** Ahmed Haleem, Phelopater Sedrak, Chetan Gohal, George S. Athwal, Moin Khan, Bashar Alolabi

**Affiliations:** 1 Faculty of Health Sciences, McMaster University, Hamilton, ON, Canada; 2 Division of Orthopaedic Surgery, Department of Surgery, McMaster University, Hamilton, ON, Canada; 3 Schulich School of Medicine & Dentistry, Western University, London, ON, Canada; 4 St. Joseph’s Healthcare Hamilton, Hamilton, ON, Canada

**Keywords:** total shoulder arthroplasty, hybrid glenoid, systematic review, polyethylene glenoid, shoulder, arthroplasty

## Abstract

**Background:**

Hybrid glenoid components in total shoulder arthroplasty (TSA) utilize both polyethylene and metal components to provide short-term stability and long-term biologic fixation through bone ingrowth.

**Questions/Purpose:**

We sought to systematically review the literature for studies that assessed outcomes of TSA performed using hybrid glenoid components.

**Methods:**

PubMed, Medline, Cumulative Index to Nursing and Allied Health Literature (CINAHL), and Embase were searched systematically for articles measuring clinical and patient-reported outcomes and rates of complication and revision following TSA using a hybrid glenoid component.

**Results:**

Seven studies with 593 shoulders were included in this review. The mean age of patients was 65 ± 1 years, and 46% of the population was male. Mean follow-up was 50 months (4.2 years). The overall complication rate was 7% and rate of revision was 2.5%; glenoid radiolucency was present in 33% of shoulders at mean follow-up of 50 months. Mean improvements in forward elevation, external rotation, internal rotation score, and abduction were 49°, 28°, 2 points, and 42°, respectively. Mean improvements in Constant, American Shoulder and Elbow Surgeons (ASES), and University of California, Los Angeles (UCLA) scores were 36 points, 52 points, and 17 points, respectively.

**Conclusion:**

Our review found that TSA using hybrid glenoid components results in low rates of complication and revision at early follow-up. Long-term studies are warranted to understand more fully the role of hybrid glenoid components in TSA.

## Introduction

Total shoulder arthroplasty (TSA) is increasingly being used as the procedure of choice for advanced shoulder pathologies, such as osteoarthritis and rheumatoid arthritis [24]. The procedure’s success depends on many factors, including patient preoperative health, severity of shoulder degeneration, integrity of the rotator cuff, and prosthesis design [24]. Neer first developed the humeral prosthesis in 1955 and improved the design in the 1970s; the surgical techniques and prostheses used continue to advance [16,17].

The glenoid component is often considered the weak link in TSA, as many failures are related to glenoid loosening [19]. Currently, the gold standard for TSA is the use of all-polyethylene glenoid components, with cementing techniques used to achieve early implant stability [11,13,22,25]. While this design offers initial stability, symptomatic glenoid loosening over time is common and may require revision surgery [19]. Metal components have demonstrated excellent outcomes in hip and knee arthroplasty, via both cemented and press-fit techniques, and this popularized the use of metal-backed glenoid components in an attempt to address the long-term concerns of all-polyethylene glenoid components [4,6]. Porous-coated, metal-backed glenoid components were designed with the goal of allowing natural bony ingrowth into the prosthesis over time to obtain long-term stability. Despite success in pain reduction and restoration of function, metal-backed components present significant complications. A 2014 systematic review by Papidonikolakis and Matsen [19] demonstrated that metal-backed glenoid components had a significantly higher rate of failure than all-polyethylene components. They also found that, while the main reason for failure in all-polyethylene components was glenoid component loosening, metal-backed components failed due to many other reasons including component fracture, metal wear, polyethylene wear, and component dissociation [19].

In light of the failure of metal-backed glenoids to properly address the long-term concerns of all-polyethylene components, hybrid glenoid components have been designed with the aim of combining the initial stability provided by cementing the polyethylene components with the long-term advantage of biologic fixation through ongrowth of metal components [8]. While there is variation in how hybrid designs are achieved, generally hybrid glenoid designs achieve fixation using elements of both polyethylene and metal components. Polyethylene components allow for initial structural stability through cement fixation and porous metal components allow for biologic fixation through bone ongrowth over time resulting in long-term stability. Examples of hybrid designs include peripheral polyethylene pegs and a central porous titanium post or polyethylene pegs with a porous metal cap. It is hypothesized that this design would reduce the incidence of glenoid component loosening seen with all-polyethylene components and would also reduce the chances of excessive polyethylene and metal wear and screw breakage seen with metal-backed components [5,18]. With this in mind, multiple studies have compared hybrid glenoid components with all-polyethylene or metal-backed ones [5,8]. Others have used hybrid components in single-intervention noncomparative studies to explore the long-term effects of this design [18].

The purpose of this systematic review was to evaluate the literature on the effects of using hybrid glenoid components in anatomic TSA on rates of failure, glenoid loosening, radiolucency, and complications. Our hypothesis was that hybrid glenoid components offered greater initial stability and had lower complication rates than all-polyethylene and metal-backed glenoid components.

## Methods

A comprehensive literature search of the PubMed, Medline, Embase, and CINAHL (Cumulative Index to Nursing and Allied Health Literature) databases was performed for relevant titles from database inception to December 1, 2019 (Supplemental Appendix Table 1). The research question and criteria for study inclusion and exclusion were determined *a priori.* Screening of titles, abstracts, and full text was done in duplicate by 2 independent reviewers (A.H., P.S.). Disagreements at the title and abstract stages were automatically carried forward to the next stage. Disagreements at the full-text stage were resolved by an independent arbitrator (C.G.). An unweighted κ statistic was calculated at each stage to assess agreement.

We applied the following inclusion criteria: each study (1) assessed outcomes following TSA, (2) assessed the use of a hybrid glenoid component, and (3) was peer reviewed and published in the English language. The exclusion criteria were (1) studies with a sample which used nonhybrid glenoid components unless outcomes for those with hybrid components were reported separately, (2) case reports, (3) biomechanical studies, (4) technique articles, and (5) review articles. If studies were suspected to have the same patient population, the study with the higher methodological quality was included.

Data were abstracted independently by the 2 reviewers (A.H., P.S.) using an electronic data abstraction form. Relevant data were abstracted including study characteristics, patient demographics, details regarding operative procedures, outcome scores, and rates of complication and failure.

The primary outcomes were rates of complication and revision, as well as radiographic findings. Secondary outcomes abstracted were pre- and postoperative values for shoulder range of motion (ROM), and patient-reported outcome measures including the Visual Analog Scale (VAS), constant score (CS), American Shoulder and Elbow Surgeons (ASES) score, University of California Los Angeles (UCLA) score, and the Shoulder Pain and Disability Index (SPADI).

Methodological quality of included studies was assessed in duplicate by both reviewers independently using the Methodological Index for Non-Randomized Studies (MINORS) tool [20]. The MINORS tool assesses nonrandomized, noncomparative studies on 8 different criteria, with a maximum of 2 points per criterion for a maximum of 16 points. Nonrandomized, comparative studies are assessed on 4 additional criteria for a maximum of 24 points. Methodological quality was categorized *a priori* as follows: a score of 0 to 8 or 0 to 12 was considered poor quality, 9 to 12 or 13 to 18 was considered fair quality, and 13 to 16 or 19 to 24 was considered excellent quality, for noncomparative and comparative studies, respectively.

### Statistics

Descriptive statistics including mean and measures of spread were used to report demographic information and outcome scores. Outcome scores were pooled where applicable. Inter-reviewer agreement for screening was assessed using an unweighted κ statistic at all screening stages. Inter-reviewer agreement for the quality assessment was evaluated using the intraclass correlation coefficient (ICC). Agreement scores were categorized *a priori* as follows: 0.81 to 0.99 was considered as almost perfect agreement; 0.61 to 0.80 was substantial agreement; 0.41 to 0.60 was moderate agreement; 0.21 to 0.40 fair agreement; and a value of 0.20 or less was considered slight agreement [12].

## Results

**Fig. 1.**
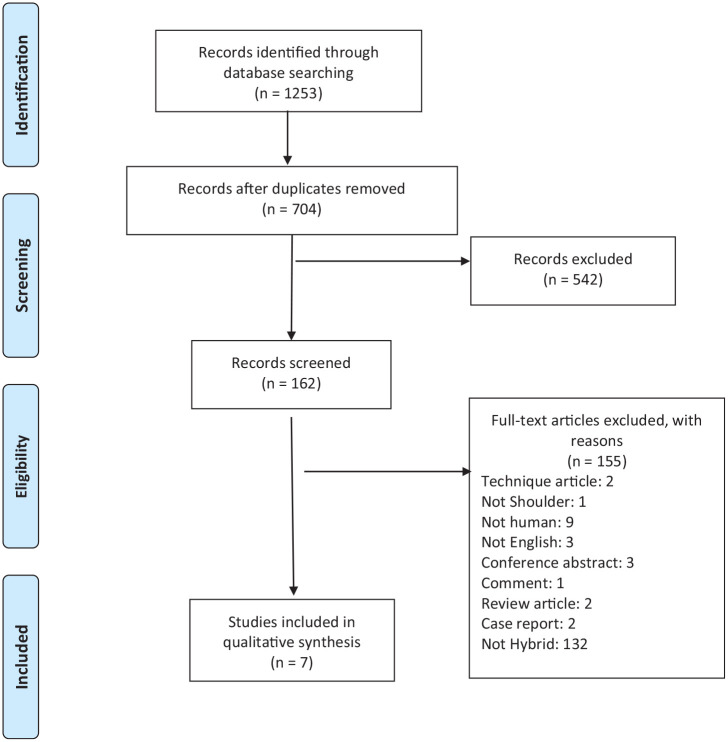
PRISMA (Preferred Reporting Items for Systematic Reviews and Meta-Analyses) flow diagram.

The literature search yielded 1253 articles. Once duplicates were removed, 704 titles remained for screening, from which 285 were included for the abstract screening. After applying inclusion and exclusion criteria, we identified 7 articles for inclusion in this review. The κ scores at the title, abstract, and full-text stages were 0.77 (95% CI: 0.72-0.82), 0.86 (95% CI: 0.80-0.92), and 0.72 (95% CI: 0.52-0.91), respectively, indicating substantial agreement at the title and full-text stages and almost complete agreement at the abstract stage (Fig. 1).

Of the 7 included articles, 3 were prospective studies and 4 retrospective studies. This included 3 cohort studies (1 prospective and 2 retrospective) and 4 case series (2 prospective and 2 retrospective). Included studies were of fair quality with a mean MINORS score of 9.6 for noncomparative studies and 15.2 for comparative studies. Inter-reviewer agreement was almost perfect with ICC = 0.84. Methodological quality and study characteristics are presented in Table 1.

**Table 1.** Study characteristics and methodological quality of included studies.

Author [year of publication]	Study type	Study design	Mean follow-up (months)	Sample size	Mean age (years)	Sex (% male)	Indication for surgery	Glenoid used	Methodological index for nonrandomized studies score
Friedman et al [5]	Retrospective	Cohort	40.5	316	65.3	44%	Osteoarthritis	Equinoxe Cage Glenoid (Exactech)	15
Nelson et al [18]	Prospective	Case series	66.5 (range: 60-84)	45	64.7 (range: 46-90)	N/R	Osteoarthritis	Comprehensive Shoulder System (Biomet, Warsaw, IN)	10
Gray et al [7]	Prospective	Cohort	25.3	46	63.2 (SD 9.4)	59%	Osteoarthritis	Equinoxe Cage Glenoid (Exactech)	15
Gulotta et al [8]	Retrospective	Cohort	38	43	66.3	47%	N/R	Comprehensive Shoulder System with Regenerex Hybrid Glenoid (Biomet)	16
Merolla et al [14]	Retrospective	Case series	38	40	63.8	60%	Osteoarthritis	Second Generation Trabecular tantalum glenoid component (Zimmer, Warsaw, IN)	11
Budge et al [2]	Prospective	Case series	38 (range: 24-64)	19	62.8 (SD 14.6)	26%	15 osteoarthritis; 1 juvenile rheumatoid arthritis; 1 rheumatoid arthritis; 1 avascular necrosis; 1 posttraumatic arthritis	Trabecular metal glenoid component (Zimmer)	12
Gurin and Seitz [9]	Retrospective	Case series	101	84	N/R	N/R	N/R	Trabecular metal glenoid (Zimmer)	7

A total of 593 shoulders were included in this study. The mean age of included patients was 65 ± 1 years. Sex was reported by 5 studies with a total of 464 participants [2,5,7,8,14]. Among these studies 46% of the population was male. The mean follow-up of included studies was 50 months (4.2 years). Hand dominance was rarely reported. The indication for surgery was osteoarthritis in 445 shoulders (62%), juvenile rheumatoid arthritis, rheumatoid arthritis, avascular necrosis and posttraumatic arthritis in 1 shoulder each (0.1%), and not reported for the remaining 271 shoulders (38%).

Various hybrid glenoid components were used. Two studies with 362 shoulders used the Equinoxe Cage Glenoid (Exactech, Inc., Gainesville, Florida) [5,7], and 2 studies with 88 shoulders used the Comprehensive Shoulder System (Zimmer Biomet, Warsaw, Indiana) [8,18]. Two studies with 103 shoulders used the trabecular metal glenoid (Zimmer Biomet) [2,9], and 1 study with 40 shoulders used the second-generation trabecular tantalum glenoid (Zimmer Biomet) [14].

The rate of complications was reported by 6 studies with a total of 548 shoulders [2,5,7–9,14]. Complications occurred in 38 shoulders giving an overall complication rate of 7%. The most common complications were rotator cuff tears in 6 shoulders (1%) and infection in 5 shoulders (1%). Regarding complications specifically related to the glenoid component, glenoid aseptic loosening and glenoid fracture each occurred in 4 shoulders (0.7%). Furthermore, there was 1 case of the polyethylene component shearing off the post at the screw-in mechanism. Other complications included articular surface dissociation, postoperative pain, nerve injuries, clavicular fractures, aseptic humeral loosening, acromioclavicular joint injuries, and hematomas.

Regarding radiolucency, 5 studies with radiographic follow-up for 351 shoulders reported the percentage of shoulders with radiolucency [2,5,7,14,18]. Of these shoulders, 33% had some degree of radiolucency.

There were 15 reported revisions in this review resulting in an overall rate of revision of 2.5%. The reasons for revision were aseptic glenoid loosening (4), articular surface dissociation (8), posterior instability (1), glenoid fractures (3) rotator cuff tear plus polyethylene wear (1), and 1 case of the polyethylene component shearing off the post at the base of the screw-in mechanism. Data on rates of complication, revision, and radiolucency are presented in Table 2.

**Table 2.** Complications, failure rates, and radiographic findings.

Author [year of publication]	Complications	Revision	Radiographic findings
Friedman et al [5]	Total: 25 (7.9%) Glenoid aseptic loosening in 4; Articular surface dissociation in 4; 4 rotator cuff tears; 4 shoulders with postoperative pain; 3 infections; 2 nerve injuries; 1 acromioclavicular joint injury; 1 clavicular fracture; 1 hematoma; aseptic humeral loosening in 1	8 (2.5%) Aseptic glenoid loosening (4); Articular surface dissociation (4)	Final follow-up available for 211 shoulders at mean follow-up of 48.4 months; Glenoid: 37.6% had some degree of glenoid radiolucency; 1.9% had radiolucency higher than grade II Distribution: 11 grade I; 4 grade II; 1 grade III; 1 grade IV; 1 grade V (Radiolucency graded according to the method of Lazarus et al [13]) Mean time to radiolucency: 37.5 ± 19.2 months Humeral: 3.0% had some degree of humeral loosening
Nelson et al [18]	1 (2.2%) Polyethylene component sheared off the post at the base of the screw in mechanism	1 (2.2%) Polyethylene component sheared off the post at the base of the screw-in mechanism	Total: 29 (64%) had some degree of radiolucency; 6 (13%) implants had radiolucency directly below glenoid faceplate; 13 (29%) had radiolucency around central post; 9 (20%) had radiolucency in 2 columns aside from the glenoid faceplate but were not judged to be at risk Mean follow-up: 66.5 months
Gray et al [7]	3 (6.5%); 1 infection; 1 adhesive capsulitis; 1 aseptic humeral loosening	N/R	Final data available for 36 shoulders; 5 shoulders (13.5%) had radiolucent lines with an average radiographic line score of 0.22’ Mean follow-up: 22.5
Gulotta et al [8]	1 (2.3%) Posterior instability	1 (2.3%) Posterior instability	Radiolucent score: 1 ± 0.4 Mean follow-up: 3.2 years
Merolla et al [14]	1 massive rotator cuff tear with static superior humeral subluxation; 1 patient showed thinning of posterior keel and wear at the polyethylene-metal interface but asymptomatic	0	Glenoid: 2 shoulders with <1 mm lines in zones 1-3; no radiographic evidence of failure Humeral: 2 shoulders with <0.5 mm lines in 2 zones; 1 patient with <1.5 mm lines in 8 zones Mean follow-up: 38 months
Budge et al [2]	Total: 5 (19%) Fractures in 4 glenoids; 1 case of shoulder instability	3 Glenoid fractures	1 component with grade II radiolucency; 4 shoulders in 4 shoulders had evidence of tantalum particulate debris or migration of the glenoid Mean follow-up: 38 months
Gurin and Seitz [9]	1 late infection; 1 case of polyethylene wear and metal debris due to rotator cuff tear	1 Polyethylene wear and metal debris due to rotator cuff tear	No loosening or implant failure noted at 8.4 years; some had radiolucency but none were loose Mean follow-up: 8.4 years

### Range of Motion

Postoperative ROM after TSA with hybrid glenoid components was reported by 6 studies [2,5,7,9,14,18], and 6 studies with 550 shoulders reported forward elevation [2,4,7,9,14,18]. Of these, 4 studies with 426 shoulders were pooled, demonstrating a mean improvement in forward elevation of 49° (range: 38°–56°) [2,5,7,18]. Five studies with 466 shoulders reported external rotation [2,5,7,14,18]. Of these, 4 studies with 426 shoulders were pooled demonstrating a mean improvement in external rotation of 28° (range: 14°–45°) [2,5,7,18]. Four studies with 447 shoulders reported internal rotation [5,7,14,18]. Of these, 2 studies with 362 shoulders were pooled, demonstrating a mean improvement in internal rotation score of 2 points (range: 1.8°–1.9°) [5,7]. Internal rotation score is an outcome measure ranging from 1 (significant deficits) to 5 (no difficulty with internal rotation tasks) developed to assess functional internal rotation after total shoulder arthroplasty [1]. Abduction was reported by 3 studies with 402 shoulders [5,7,14]. Of these, 2 studies with 362 shoulders were pooled, demonstrating a mean improvement in abduction of 42° (range: 31.1°–43.5°) [5,7]. Not all studies were pooled due to heterogeneity in measurement of ROM.

Statistically significant improvements were reported in forward elevation by 2 studies [14,18], external rotation by 2 studies [14,18], internal rotation by 2 studies [14,18], and abduction by 1 study [14]. Among studies which did not comment on statistical significance, substantial improvements were demonstrated in ROM by all other studies. Range of motion results are presented in Table 3.

**Table 3.** Range of motion.

Author [year of publication]	Forward elevation (degrees)	External rotation (degrees)	Internal rotation (degrees unless otherwise indicated)	Abduction (degrees)
Friedman et al [5]	Pre-op: 100.1 (SD: 34.6) Post-op: 150 (SD: 30.4) Improvement: 50.7 (SD 41.8)	Pre-op: 22.2 (SD: 18.8) Post-op: 50.7 (SD: 20.6) Improvement: 29.2 (SD 22.2)	Pre-op: 3.2 (SD: 1.6) Post-op: 5.0 (SD: 1.4) Improvement: 1.9 (SD 1.7) Measured as internal rotation score	Pre-op: 88.2 (SD: 33.6) Post-op: 130.6 (SD: 37.2) Improvement: 43.5 (SD: 46.0)
Nelson et al [18]	Pre-op: 113 Post-op: 151 Improvement: 38 *P* < .001	Pre-op: 50 Post-op: 36 Improvement: 14 *P* < .001	Pre-op: 49 Post-op: 60 Improvement: 11 *P* < .05	N/R
Gray et al [7]	Pre-op: 104.4 (SD: 32.8) Post-op: 148.9 (SD: 23.8) Improvement: 45.1 (SD: 35.9)	Pre-op: 22.4 (SD: 19.3) Post-op: 49.1 (SD: 18.1) Improvement: 26.4 (SD: 17.1)	Pre-op: 3.5 (SD: 1.5) Post-op: 5.2 (SD: 1.2) Improvement: 1.8 (SD: 1.7) Measured as internal rotation score	Pre-op: 93.1 (SD: 30.3) Post-op: 124.3 (SD: 29.1) Improvement: 31.1 (SD: 32)
Merolla et al [14]	Pre-op: 5.4 (SD: 2.1) Post-op: 9.4 (1.5) *P* < .05 *2 points assigned to every 30° of movement	Pre-op: 2.3 (SD: 0.7) Post-op: 4.5 (SD: 3.5) *P* < .001 *measured from 0-10 points; 2 points = hand behind head with elbow forward; 15 points = complete range of motion	Pre-op: 3.3 (SD: 1.5) Post-op: 7.2 (SD: 1.9) *P* < .001 *measured from 0-10 points; 0 = dorsum of hand to lateral thigh; 10 = dorsum of hand to interscapular region	Pre-op: 4.7 (SD: 1.7) Post-op: 9.0 (SD: 1.4) *P* < .001 *2 points assigned to every 30° of movement
Budge et al [2]	Pre-op: 75 (range: 20-126) Post-op: 131 (range: 80-170) Improvement: 56 (SD 34)	Pre-op: 5 (range: -25-30) Post-op: 49 (range: 0-60) Improvement: 45 (SD 29.8)	N/R	N/R
Gurin and Seitz [9]	Overhead range of motion improved from 62° (pre-op) to 145° (post-op)	N/R	N/R	N/R

### Patient-Reported Outcomes

Various patient-reported outcomes were reported. Constant score was reported by 3 studies with 402 shoulders [5,7,14]. Mean improvement in Constant score was 36 points (range, 35-47). Two studies with 362 shoulders reported UCLA scores and demonstrated a mean improvement of 17 points (range: 16.9-17.1) [5,7]. Six studies with 509 shoulders reported ASES and demonstrated a mean improvement of 52 points (range: 39-70) [2,5,7,8,14,18]. SST was reported by 2 studies with 362 shoulders with a mean improvement of 7 points (range: 6.6-6.7) [5,7]. SPADI was reported by 2 studies with 362 shoulders with a mean improvement of 69 points (range: 67-69) [5,7]. VAS was reported by 4 studies with 212 shoulders with a mean improvement of 6 points (range: 5-7) [2,8,9,18].

Statistically significant improvements were found in Constant score by 1 study [14], ASES by 3 studies [2,14,18], and VAS by 2 studies [2,18]. One study reported pain on a scale of 1 to 5 and also found statistically significant improvements [14]. Among studies that did not comment on statistical significance, substantial improvements were found in all patient-reported outcome measures (Table 4).

**Table 4.** Patient-reported outcome measures.

Author [year of publication]	Constant score	UCLA score	ASES score	SST	SPADI	VAS
Friedman et al [5]	Pre-op: 37.8 (SD: 15.0) Post-op: 73.6 (SD: 14.6) Improvement: 35.1 (15.0)	Pre-op: 14 (SD: 4) Post-op: 30.8 (SD: 5.8) Improvement: 16.9 (5.8)	Pre-op: 34.6 (SD: 16.6) Post-op: 86.7 (SD: 18.6) Improvement: 53.3 (SD: 21.9)	Pre-op: 4 (SD: 3) Post-op: 10.7 (SD: 2.4) Improvement: 6.7 (SD: 3.4)	Pre-op: 84.2 (SD: 25.2) Post-op: 15.7 (22.8) Improvement: 69.2 (SD: 30)	N/R
Nelson et al [18]	N/R	N/R	Pre-op: 40.4 Post-op: 83.7 Improvement: 43.3 (*P* < .0001)	N/R	N/R	Pre-op: 5.9 Post-op: 0.8 Improvement: 5.1 (*P* < .001)
Gray et al [7]	Pre-op: 38.5 (SD: 15.9) Post-op: 75.7 (SD: 13.5) Improvement: 36.3 (SD: 12.6)	Pre-op: 15.4 (SD: 4.1) Post-op: 31.8 (SD: 4.3) Improvement: 17.1 (SD: 4.4)	Pre-op: 37.3 (SD: 18.8) Post-op: 89.3 (SD: 16.3) Improvement: 51.7 (SD: 18.7)	Pre-op: 4.1 (SD: 3.1) Post-op: 10.8 (SD: 2.2) Improvement: 6.6 (SD 3.1)	Pre-op: 77.9 (SD: 25.8) Post-op: 11.1 (SD: 17.6) Improvement: 67.3 (SD 26.7)	N/R
Gulotta et al [8]	N/R	N/R	Pre-op: 35.9 (SD 7.1) Post-op: 83.5 (SD 13.1) Improvement: 38.7 (SD: 7.3)	N/R	N/R	Pre-op: 7.1 (SD 2.1) Post-op: 1.2 (SD 0.2) Improvement: 5.8 (SD 0.7)
Merolla et al [14]	Pre-op: 23.2 (SD: 6.4) Post-op: 69.8 (SD: 13.2) *P* < .001	N/R	Pre-op: 24.1 (SD: 10.7) Post-op: 93.4 (SD : 6.8) *P* = .009	N/R	N/R	*pain on scale of 0-15, with 15 being no pain Pre-op: 2.1 (SD: 2.3) Post-op: 14.6 *P* < .001
Budge et al [2]	N/R	N/R	Pre-op: 21.3 (range: 0-42) Post-op: 70.5 (range: 50-100) *P* < .05	N/R	N/R	Pre-op: 8.6 (range: 5-10) Post-op: 2.9 (range: 0-5) *P* < .001
Gurin and Seitz [9]	N/R	N/R	N/R	N/R	N/R	Pre-op: 8.2 Post-op: 1.1

*UCLA* University of California, Los Angeles, *ASES* American Shoulder and Elbow Surgeons, *SPADI* Shoulder Pain and Disability Index, *VAS* Visual Analog Scale, *SST* Simple Shoulder Test.

The overall rate of complication in this study was 7% at a mean follow-up of 50 months. Gray et al [7] followed up 96 shoulder arthroplasties at mean follow-up of 26 months, Gulotta et al [8] followed 83 shoulder arthroplasties at 38 months, and Friedman et al [5] followed 632 at 41 months. All 3 studies found equivalent rates of complications in hybrid and all-polyethylene glenoid components. Regarding the rate of revision, Gulotta et al [8] found an identical rate of revision between the 2 groups, while Friedman et al [5] found a reduced rate of revision in the hybrid glenoid group (3% vs 7%; *P* = .0088). Regarding radiolucency, Friedman et al [5] found significantly fewer cases of radiolucency in the hybrid glenoid group (9% vs 38%; *P* < .001) and Gulotta et al [8] found a lower radiolucency score in the hybrid group, but the difference did not reach statistical significance (1.0 vs 1.6; *P* = .323). Finally, Gray et al [7] did not report on statistical significance but found 50% fewer cases of radiolucency (14% vs 28%) and a 50% lower radiolucency score (0.2 vs 0.6) in the hybrid glenoid group. Taken together, the evidence suggests that rates of complication and revision are equivalent between hybrid and polyethylene glenoid components. However, rates of radiolucency seem to be higher in all-polyethylene glenoid components. As the titanium post in hybrid glenoids is designed to provide long-term biologic fixation, it may take longer follow-up periods for differences in failure rates between the 2 glenoid designs to be seen. As for rates of radiolucency, poor cementing technique and component instability have both been attributed as causes for radiolucency [23]. Biomechanical studies have demonstrated increased initial stability of hybrid glenoid components due to the central peg design [3], which may explain the reduced rate of radiolucency.

## Discussion

This systematic review of the literature on outcomes following anatomic TSA using hybrid glenoid components found rates of complication and revision are quite good, at 7% and 2.5%, respectively, at an average follow-up of 50 months. Furthermore, consistent improvements were demonstrated in ROM and patient-reported outcome measures. Noteworthy strengths of this study include its a rigorous screening process performed in duplicate to minimize reviewer bias, with excellent agreement between reviewers at all screening stages.

There are also some limitations to this systematic review. First, as these implants are relatively new, the average follow-up is only 50 months, and longer follow-up will be required to determine differences in implant survivorship. Second, the indication for surgery for almost all included patients was osteoarthritis. As such, while the results of this review may be most appropriate for that population, they may not be applicable to patients with other indications for TSA. Third, there were different measurement techniques used for some outcome measures making it impossible to pool all data. Lastly, most studies were of low-level evidence and were noncomparative. Further high-quality, comparative studies are necessary to more completely understand the role of hybrid glenoid components in TSA.

Overall, studies reported substantial improvements in ROM and patient-reported outcomes. Improvements in Constant, ASES, UCLA, SST, and VAS scores were all clinically significant based on previously reported minimal clinically important differences [10,21,26]. Improvements in forward elevation, external rotation, and abduction were also all clinically significant, though statistical significance was not always reported [15]. Regarding comparisons to nonhybrid glenoid components, 3 cohort studies compared TSA performed with hybrid glenoid components and all-polyethylene glenoid components; all found that improvements in ROM and patient-reported outcome scores were not inferior to those found in all-polyethylene glenoid components [5,7,8].

It is worth noting that the rates of radiolucency did not relate consistently with rates of revision or loosening. Gulotta et al [8] found no difference in rates of radiolucency and revision, and Friedman et al [5] found lower radiolucency and revision rates in the hybrid glenoid group. However, Gray et al [7] in 2015 found 50% lower radiolucency but no difference in rates of revision. As such, the clinical significance of radiolucency is unclear. However, longer term follow-up would be required to more accurately determine if decreased radiolucency would lead to decreased loosening or revision.

A systematic review assessing 1571 metal-backed glenoid components and 3035 all-polyethylene components at 6 to 7 years of follow-up demonstrated rates of revision for metal-backed and all-polyethylene components to be 14% and 4%, respectively [19]. Rates of radiographic loosening or failure for metal-backed and all-polyethylene components were 21 and 17%, respectively, and rates of radiolucency were 35% and 42%, respectively [19]. These are higher than the rates found in our review. However, the review also found that rates of revision increased significantly in studies with longer follow-up, particularly after 7 years. The mean follow-up was 6 years for all-polyethylene components and 7 years for metal-backed components. As the follow-up in this review was much lower, it is difficult to draw conclusions from these comparisons.

In conclusion, or review of level III and level IV studies found that anatomic TSA using a hybrid glenoid component results in excellent improvements in ROM and patient-reported outcomes; rates of complication and revision were comparable to TSA using a conventional, all-polyethylene glenoid component. High-quality studies with long-term follow-up are required to determine if hybrid glenoid components hold significant advantage over conventional all-polyethylene glenoid components in TSA.

## Supplemental Material

sj-docx-1-hss-10.1177_15563316211040829 – Supplemental material for Hybrid Glenoid Designs in Anatomic Total Shoulder Arthroplasty: A Systematic Review Click here for additional data file. Supplemental material, sj-docx-1-hss-10.1177_15563316211040829 for Hybrid Glenoid Designs in Anatomic Total Shoulder Arthroplasty: A Systematic Review by Ahmed Haleem, Phelopater Sedrak, Chetan Gohal, George S. Athwal, Moin Khan and Bashar Alolabi in HSS Journal®: The Musculoskeletal Journal of Hospital for Special Surgery

sj-pdf-2-hss-10.1177_15563316211040829 – Supplemental material for Hybrid Glenoid Designs in Anatomic Total Shoulder Arthroplasty: A Systematic Review Click here for additional data file. Supplemental material, sj-pdf-2-hss-10.1177_15563316211040829 for Hybrid Glenoid Designs in Anatomic Total Shoulder Arthroplasty: A Systematic Review by Ahmed Haleem, Phelopater Sedrak, Chetan Gohal, George S. Athwal, Moin Khan and Bashar Alolabi in HSS Journal®: The Musculoskeletal Journal of Hospital for Special Surgery

sj-pdf-3-hss-10.1177_15563316211040829 – Supplemental material for Hybrid Glenoid Designs in Anatomic Total Shoulder Arthroplasty: A Systematic Review Click here for additional data file. Supplemental material, sj-pdf-3-hss-10.1177_15563316211040829 for Hybrid Glenoid Designs in Anatomic Total Shoulder Arthroplasty: A Systematic Review by Ahmed Haleem, Phelopater Sedrak, Chetan Gohal, George S. Athwal, Moin Khan and Bashar Alolabi in HSS Journal®: The Musculoskeletal Journal of Hospital for Special Surgery

sj-pdf-4-hss-10.1177_15563316211040829 – Supplemental material for Hybrid Glenoid Designs in Anatomic Total Shoulder Arthroplasty: A Systematic Review Click here for additional data file. Supplemental material, sj-pdf-4-hss-10.1177_15563316211040829 for Hybrid Glenoid Designs in Anatomic Total Shoulder Arthroplasty: A Systematic Review by Ahmed Haleem, Phelopater Sedrak, Chetan Gohal, George S. Athwal, Moin Khan and Bashar Alolabi in HSS Journal®: The Musculoskeletal Journal of Hospital for Special Surgery

sj-pdf-5-hss-10.1177_15563316211040829 – Supplemental material for Hybrid Glenoid Designs in Anatomic Total Shoulder Arthroplasty: A Systematic Review Click here for additional data file. Supplemental material, sj-pdf-5-hss-10.1177_15563316211040829 for Hybrid Glenoid Designs in Anatomic Total Shoulder Arthroplasty: A Systematic Review by Ahmed Haleem, Phelopater Sedrak, Chetan Gohal, George S. Athwal, Moin Khan and Bashar Alolabi in HSS Journal®: The Musculoskeletal Journal of Hospital for Special Surgery

sj-pdf-6-hss-10.1177_15563316211040829 – Supplemental material for Hybrid Glenoid Designs in Anatomic Total Shoulder Arthroplasty: A Systematic Review Click here for additional data file. Supplemental material, sj-pdf-6-hss-10.1177_15563316211040829 for Hybrid Glenoid Designs in Anatomic Total Shoulder Arthroplasty: A Systematic Review by Ahmed Haleem, Phelopater Sedrak, Chetan Gohal, George S. Athwal, Moin Khan and Bashar Alolabi in HSS Journal®: The Musculoskeletal Journal of Hospital for Special Surgery

sj-pdf-7-hss-10.1177_15563316211040829 – Supplemental material for Hybrid Glenoid Designs in Anatomic Total Shoulder Arthroplasty: A Systematic Review Click here for additional data file. Supplemental material, sj-pdf-7-hss-10.1177_15563316211040829 for Hybrid Glenoid Designs in Anatomic Total Shoulder Arthroplasty: A Systematic Review by Ahmed Haleem, Phelopater Sedrak, Chetan Gohal, George S. Athwal, Moin Khan and Bashar Alolabi in HSS Journal®: The Musculoskeletal Journal of Hospital for Special Surgery
