# Supplementary material for: Hybrid Glenoid Designs in Anatomic Total Shoulder Arthroplasty: A Systematic Review
Source: HSS J. 2021 Sep 3;18(2):219–28. doi: 10.1177/15563316211040829 (PMC9096999; doi:10.1177/15563316211040829)
Supplement: sj-docx-1-hss-10.1177_15563316211040829 – Supplemental material for Hybrid Glenoid Designs in Anatomic Total Shoulder Arthroplasty: A Systematic Review [file sj-docx-1-hss-10.1177_15563316211040829.docx]

**Appendix Table 1.**

| **PubMed (548 results)** | **Medline (263 results)** | **Embase (369 results)** | **CINAHL (72 results)** |
| --- | --- | --- | --- |
| 1. Total shoulder arthroplasty 2. Shoulder replacement 3. Hybrid 4. Hybrid glenoid 5. Metal 6. Titanium 7. Glenoid components 8. 1 OR 2 9. 3 OR 4 OR 5 OR 6 OR 7 10. 8 AND 9 | 1. Total shoulder arthroplasty.mp 2. Shoulder replacement.mp 3. Hybrid.mp 4. Hybrid glenoid.mp 5. Metal.mp 6. Titanium.mp 7. Glenoid components.mp 8. 1 OR 2 9. 3 OR 4 OR 5 OR 6 OR 7 10. 8 AND 9 | 1. Total shoulder arthroplasty.mp 2. Shoulder replacement.mp 3. Hybrid.mp 4. Hybrid glenoid.mp 5. Metal.mp 6. Titanium.mp 7. Glenoid components.mp 8. 1 OR 2 9. 3 OR 4 OR 5 OR 6 OR 7 10. 8 AND 9 | 1. Total shoulder arthroplasty 2. Shoulder replacement 3. Hybrid 4. Hybrid glenoid 5. Metal 6. Titanium 7. Glenoid components 8. 1 OR 2 9. 3 OR 4 OR 5 OR 6 OR 7 10. 8 AND 9 |
